# Value of CT-MRI fusion in iodine-125 brachytherapy for high-grade glioma

**DOI:** 10.18632/oncotarget.22844

**Published:** 2017-12-01

**Authors:** Yang Gao, Yan Han, Guo Nan, Man Hu, Xiaobin Zhou, Xiaokun Hu

**Affiliations:** ^1^ School of Instrumentation Science and Opto-Electronics Engineering, Beihang University, Beijing 100191, China; ^2^ Department of Radiology, The Affiliated Hospital of Qingdao University, Qingdao 266001, China; ^3^ Department of Radiation Oncology, Shandong Cancer Hospital and Institute, Jinan 250117 China; ^4^ Department of Epidemiology and Health Statistics, Public Health College, Qingdao University, Qingdao 266021, China; ^5^ Interventional Center, The Affiliated Hospital of Qingdao University, Qingdao 266001, China

**Keywords:** brachytherapy, image fusion, MRI, CT, high-grade glioma

## Abstract

**Purposes:**

To develop a fast, accurate and robust method of fusing Computed Tomography (CT) with pre-operative Magnetic Resonance Imaging (MRI) and evaluate the impact of using the fused data on the implantation of Iodine-125 (^125^I) seeds for brachytherapy of high-grade gliomas (HGG).

**Methods:**

A study was performed on a cohort of 10 consecutive patients with HGG were treated by ^125^I brachytherapy with CT-MRI fusion image guided (CMGB), and 10 patients treated with CT alone guided (CGB). Statistical analysis was performed to compare (1) the planning target volume, (2) the accuracy of location of catheters, (3) the target volume covered by 150% prescribe dose (*V150*), (4) the target volume covered by 200% prescribe dose (*V200*), and (5) the conformity index (*CI*) with or without fused data.

**Results:**

The median planning target volume was 50.1 cm^3^ in CGB, and 56.25 cm^3^ in CMGB with significant difference (*p* = 0.005). The accuracy of catheter insertion was 94.4% with CMGB and 78.9% with CGB. The median *V150* and *V200* was 45.32% vs 64.24% and 32.81% vs 53.17% in CGB and CMGB, respectively. There was significant difference for *CI* (83.5% *vs.* 74.5%, *p* < 0.05) in the two groups for the post-operative verification.

**Conclusions:**

The proposed MRI-CT fusion method enables a quantitative assessment of impact on HGG brachytherapy. The additional information obtained from the fused images can be utilized for more accurate delineation of lesion boundaries and targeting of catheters. Experimental results show that the fusion algorithm is robust and reliable in clinical practice.

## INTRODUCTION

High-grade glioma (HGG) is the most common malignant histology and aggressive in nature of gliomas. With the standard treatment of total surgical resection followed by radiotherapy and adjuvant chemotherapy, the median overall survival time (OS) HGG patients is only 12-18 months [1]. Local failure is a major concern for patients with HGG, which leads to a poor OS. Brachytherapy as the sole treatment for HGG is highly controversial, and has been a subject of dispute ever since its implementation. Recently several retrospective and prospective studies showed Iodine-125 (^125^I) seeds implanted brachytherapy is a safe, minimally invasive and effective local control treatment option for patients with recurrent and pre-irradiated HGG [2–5]. Brachytherapy is a feasible option for improved overall survival in carefully selected patients with HGG.

Modern seed implantation brachytherapy evolved with increases in computational power for dose planning and catheter targeting. Typically, preoperative CT is used for treatment planning and intra-operative CT is used for the guidance of seed implantation CT which provides excellent catheter and seed visualization, but the tissue characterization of brain anatomy is limited [6]. The lack of accurate delineation of HGG boundaries adversely affects the accuracy of the planning target volume and therefore the distribution of seeds and dosimetry [7]. In contrast, Magnetic Resonance Imaging (MRI) provide the better contrast for differentiating soft tissue. However, seed and catheter localization in MRI images is challenging [8, 9]. Besides, its relative sensitivity to movement in MRI. Combining different modalities through image fusion offsets the weakness of each modality. Therefore, the CT-MRI fusion method has thus been proposed [10]. This method can bring together the strengths of these complementary imaging modalities and provide accurate comparison of the treatment planning.

In the past decade, a growing number of investigators have reported the planning process and CT, MRI or fused image guided impanation ensure that seed placement can be optimized to deliver the prescribed dose. Several authors have analyzed fusion-based post-implant assessment of prostate brachytherapy and intra-operative CT-CT fusion for brain tumor brachytherapy [11–13]. However, there is no study comparing two series of patients with HGG treated with or without image fusion in treatment planning and intra-operative seed implanting.

This study investigated the accuracy of fusion of ^125^I seed implantation with the use of fused CT and MR images. We compared the results of planning target volume, the accuracy of location of catheters, *V150*, and *V200* with or without fused data. The study was approved by the Ethics Board of Affiliated Hospital of Qingdao University, China.

## RESULTS

Before the operation, the *PTV* was compared between pre-operative CT and CT/MRI fusion (Table 1). The median *PTV* measured on CT/MRI fusion images and CT images only was 56.25 cm^3^ and 50.1 cm^3^, respectively. There was a significantly different on delineation of the target volume between CT based and fusion-based target volume (*p* < 0.05).

**Table 1:** Delineation of the target volume between delineated under CT-MRI fused data and CT data alone

	Delineated under CT images			Delineated under CT-MRI fused data			*p*-Value	Z
	Median (Q)	Mean	Range	Median (Q)	Mean	Range		
*TV (cm^3^)*	35.25 (48.62)	45.61	9.8 - 93.5	35.25 (48.62)	45.61	9.8 - 93.5	-	-
*PTV (cm^3^)*	50.1 (58.68)	65.3	18.7 - 127.8	56.25 (60.1)	71.36	21.2 - 138.2	0.005	-2.803

Abbreviations: TV = target volume, PTV = planning target volume, Q = quartile range.

After the operation, the ***V_pd_*** , *V150* and *V200* were calculated. There was a significant different between the two groups for ***V_pd_*** , *V150* and *V200.* The median *V150* and *V200* was 45.32% vs 64.24% and 32.81% vs 53.17% in CGB and CMGB, respectively. The median conformity index was 83.5% and 74.5% respectively. The *CI* in CMGB group was higher compared to *CI* in CGB group. *CI* comparison displays 9% difference between two groups with statistical significance (*p*=0.003). Table 2 shows the physical factors appraised in the post-operative verification.

**Table 2:** Physical factors appraised in the post-operative verification

	CMGB group		CGB group		Z	*p*-Value	S
	Median (Q)	Range	Median (Q)	Range			
***Vpd (%)***	91.35 (2.23)	89.10 - 94.70	58.79 (4.00)	55.22 – 62.15	-3.787	0.00015	S
***V150(%)***	64.10 (16.88)	47.40 - 78.50	45.32 (4.47)	34.88 – 49.30	-3.63	0.00028	S
***V200(%)***	52.55 (3.65)	47.40 - 59.30	32.81 (8.24)	27.34 – 42.98	-3.787	0.00015	S
***CI (%)***	85 (4)	79 - 90	75 (7)	66 - 84	-2.926	0.003	S

Abbreviations: Vpd = prescribed dose covering the clinical target volume, TV = the target volume, V150 = the target volume covered by 150% prescribe dose, V200 = the target volume covered by 200% prescribe dose, CI = conformity index. Q = quartile range, S = significance, N = no significance.

In CMGB group, a total 107 catheters and 527 seeds were used. And 76 catheters and 380 seeds were used in CGB group. Because of the limitation of intra-operative CT images, tumor boundaries were unclear boundary in the CGB group. The post-operative verification shows that 16 catheters (21.1%) did not reach the planning point in CGB group. In CMGB group, only 6 (5.6%) catheters needed to be adjusted. The result shows that the accuracy of catheter insertion was 94.4% with CMGB and 78.9% with CGB.

## DISCUSSION

HGG is the most common primary central nervous system malignancy, treatment for HGG remains one of the greatest clinical challenges despite recent advances in extensive surgical resection, radiation therapy and chemotherapy. However, surgical resection for patients with HGG in highly eloquent areas, it was impossible to complete resection, and maybe surgery had high risk of neurologic injury. Although radiation therapy and chemotherapy are intended to target dividing tumor cells, there are normal cells in the brain which are also dividing. Furthermore, the impact of surgery and chemotherapy results also more limited in HGG. Brachytherapy for HGG was introduced in the end of last century, and has high local efficacy and comparably low morbidity.

We presented the application of an image fusion technique to intra-operative brachytherapy for patients with HGG. The CT-guided brachytherapy techniques address the limitation in the use of CT alone in surgical navigation due to the lack of tumor boundary discrimination. Our study showed delineation of the target volume on CT image and CT-MRI fused data in pre-operative treatment planning, and there was no significant difference between the two approaches. In order to improve the clinical accuracy of diagnosis, the additional information could be obtained by CT-MRI image fused data for treatment planning (Table 1).

During the past decades, a large body of work has been published on information fusion techniques [14]. Earlier studies reported the clinical application of image fusion for brain images from Positron Emission Tomography (PET), Single-Photon Emission Computed Tomography (SPECT), CT, Ultrasound (US) and MRI [10, 15–17].

Image fusion techniques performed in a broad range of applications in tumors resection, brachytherapy and radiotherapy [8–10, 12, 13, 16–36] for prostate, neck and head, lung, liver, and breast. Several studies focus on image fusion guided in brachytherapy of brain tumors [11, 13, 37, 38]. In most of these studies, however, commercial software was used and only the results of the operation were reported. Julow et al [11] demonstrated the application of intra-operative CT-CT fusion, and the use of CT-MRI and CT-PET fusion for follow-up. Although, the fusion was done for the purpose of intra-operative dosimetry evaluation, this method can also be used to check the position of catheter. In other studies [19, 37, 38], the feasibility of fusion of different imaging modalities was reported for follow-up. In our study, the algorithm was specially developed to make the CT-MRI fusion into a quality-control tool that can be used to monitor the implant process during HGG treatment.

This study demonstrated the application of the CT-MRI fusion method for planning a monitoring the operation. The higher *CI* rate with CMGB in the post-operative verification suggests that the tumor volume received closer to the ideal treatment dose while sparing normal tissue received compared to CGB patients (Table 2). CT-MRI image fusion in the operation was useful to determine the border of glioma and can aid in adjusting the position of the catheters and seeds in real time. The image fusion protocol is an essential component of our intra-operative treatment and postoperative dose verification effort. The image fusion method was used in the majority of patients treated with ^125^I seeds implanted brachytherapy, and will completely replaced the used of CT guided brachytherapy in Affiliated Hospital of Qingdao University.

This study has several limitations. The number of patients must be expanded to more fully evaluate the potential for the fusion method. Since brachytherapy was utilized as an option of treatment for HGG in most studies, we note that an outcome analysis must be performed to compare brachytherapy HGG patients to HGG patients receiving surgery, radiation, and chemotherapy.

## MATERIALS AND METHODS

### Patients

Patients with newly diagnosed HGG were enrolled between May 2002 and December 2016 in Affiliated Hospital of Qingdao University, China. The tumor was either biopsy proven or diagnosis was based on advanced MR imaging. Before brachytherapy was performed, all patients received standard pre-treatment evaluations including conventional physical examination and contrast enhance CT and MRI. Whenever necessary, SPECT and additional FDG PET/CT scan were taken. Patients were selected for interstitial brachytherapy protocol if they fulfilled the following inclusion criteria: lesion diameters ≤50 mm, lesion numbers ≤3, the survival period ≥6 months and Karnofsky performance score (KPS) ≥60. The criterial were set based on previous experience in our hospital. The exclusion criteria were major organ dysfunction, acute or chronic infections, severe organ and coagulation dysfunction, patients with brain edema and cerebral hernia, mental disorder or has a history of mental illness, and distant metastasis, and pregnancy. There were 20 patients who were to receive current brachytherapy were eligible in the study. All cases were reviewed by two experienced neurosurgeons confirmed to be suitable for brachytherapy. Patient and tumor characteristics were summarized in Table 3.

**Table 3:** Patients baseline treatment characteristics

	CMGB	CGB
No. of patients	10	10
Age (*years*)		
Mean ±SD	60.9 ± 14.29	54.2 ± 19.32
Range	24 - 73	16 - 78
Sex (*%*)		
Male	10	40
Female	90	60
Histology (WHO)	HGG	HGG
Prescribe dose (*Gy*)		
Mean ±SD	104 ± 11.74	103 ± 14.18
Range	80 - 120	80 - 120
**Tumor Volume (*cm^3^*)**		
Median	35.25	29.3
Range	9.8 - 93.5	9.8 - 63.3
Seeds implanted (*n*)		
Mean ±SD	52.7 ± 24.86	38 ± 14.18
Range	20 - 95	20 - 60
Catheters (*n*)		
Mean ±SD	10.7 ± 4.9	7.6 ± 2.84
Range	4 - 19	4 - 12
Seeds activity (*mCi*)		
Mean ±SD	0.674 ± 0.058	0.71 ± 0.09
Range	0.6 - 0.75	0.6 - 0.8
Tumor localization (*%*)		
eloquent cortical/subcortical	40	70
basal ganglia	30	10
brainstem	0	10
Other	30	10

^125^I seeds (Model 6711, 4.5 mm long, 0.8 mm in diameter, half value of 0.025mm in lead, half-life was 59.4 days, Beijing Atom and High Technique Industries Inc., Beijing, China) were implanted using a Mick applicator. A total of 907 seeds sources were implanted in the 20 study patients. The median seed activity was 0.7 mCi (0.6-0.75 mCi), and a prescribed dose (PD) of 100-130 Gy was administered for patients treated with CT-MRI fusion guided brachytherapy. One week before treatment, treatment planning was performed by neurosurgeon and supported by medical physicist (Stereotactic 3D Treatment planning software was developed by Beijing Astro Technology Ltd. Co.).

### Procedure

In order to evaluated the quality and robustness of planning and guidance with fused images. A control group was established using the treatment method already established at our institution with an experimental group. The former group is comprised of ten patients treated with CT-guided brachytherapy (CGB group). The latter group is comprised of ten patients treated with the CT-MRI fusion guided brachytherapy (CMGB group). All patients were treated by two primary surgeons and treatment planning was made by the same physicist within a period of consecutive months.

#### Pre-operation

Before the operation, CT (GE Lights peed 16 CT scanner, 5mm slice thickness. 120 kV, 250-350 mAs, the contrast material injection rate was 3.0 ml/s) and MR (GE Signa HDx, Milwaukee, WI, USA) scans were required. Images for the CMGB group were fused in fusion software which we developed, and then transfer to the Treatment Planning Software (TPS, Beijing Astro Technology Ltd. Co.). Images for the CGB group were sent directly to the TPS. The objective of radiation treatment planning for brain tumors in TPS is to determine a seeds configuration with as few seed catheters as possible (to minimize operative risk) and to achieve an optimal conformation of the therapeutic dose with respect to the surface of the target volume.

**Figure 1:**
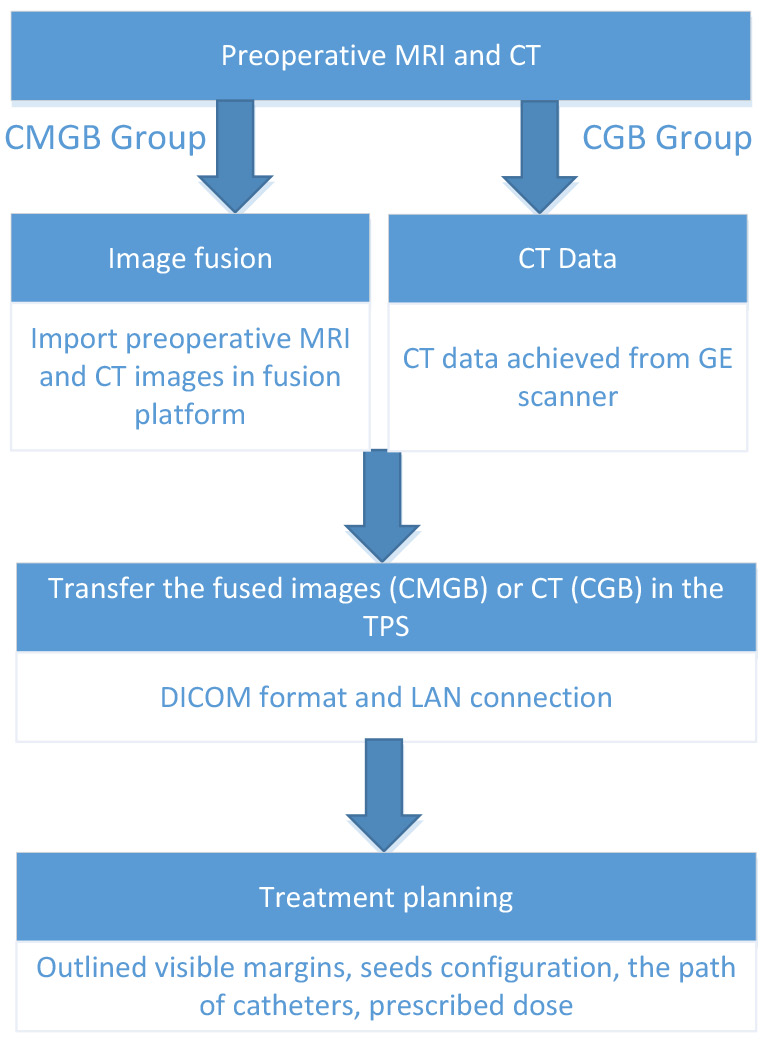
Pre-operation workflow

The desired surface dose, implantation time, and trephination point(s) are selected manually and a seed configuration yielding optimal coverage of the tumor with the prescribed dose is calculated automatically by minimization of an appropriate objective function. Figure 1 summarizes the pre-operative workflow.

**Figure 2:**
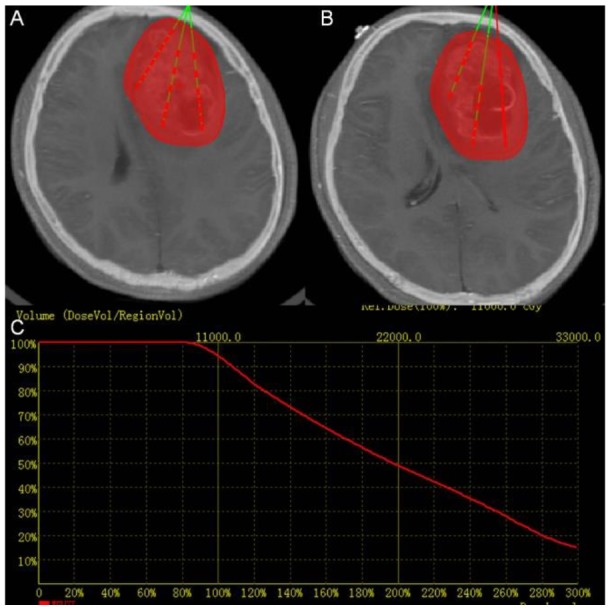
Treatment planning **(A)** and **(B)** Two slices of CT-MRI fused images showing the PTV in red and planned catheter and seed placement in green **(C)** Dose volume histogram of PTV of pre-operation treatment planning. The red curve shows the dose volume histogram of the target.

The example of a 24 years old female patient with HGG in the cerebral frontal lobe is used to illustrate the process. Visible margins of the tumor are outlined manually, and inverse treatment planning [39] is used (Figure 2).

#### Intra-operation

Intra-operative CT-imaging has the advantage of being less susceptible to distortions, while MR imaging provides better structural resolution of brain and tumor tissue. CT was performed intra-operatively, and intra-operative CT scans are fused with pre-operative MR images. Depending on the image fusion software, image fusion can be performed either automatically or by using anatomical landmarks.

The ^125^I seeds were introduced into silicon catheters. After skin incision and placement of an 8 mm burr hole, the catheters were inserted into the tumor. In case histology is requested, a stereotactic biopsy can be taken and evaluated during the operation. To ensure correct placement of the seed(s) intra-operatively we performed CT imaging in two planes (anterior/posterior and lateral) with a stationary stereotactic X-ray source, and fused these images with pre-operative MRI.

**Figure 3:**
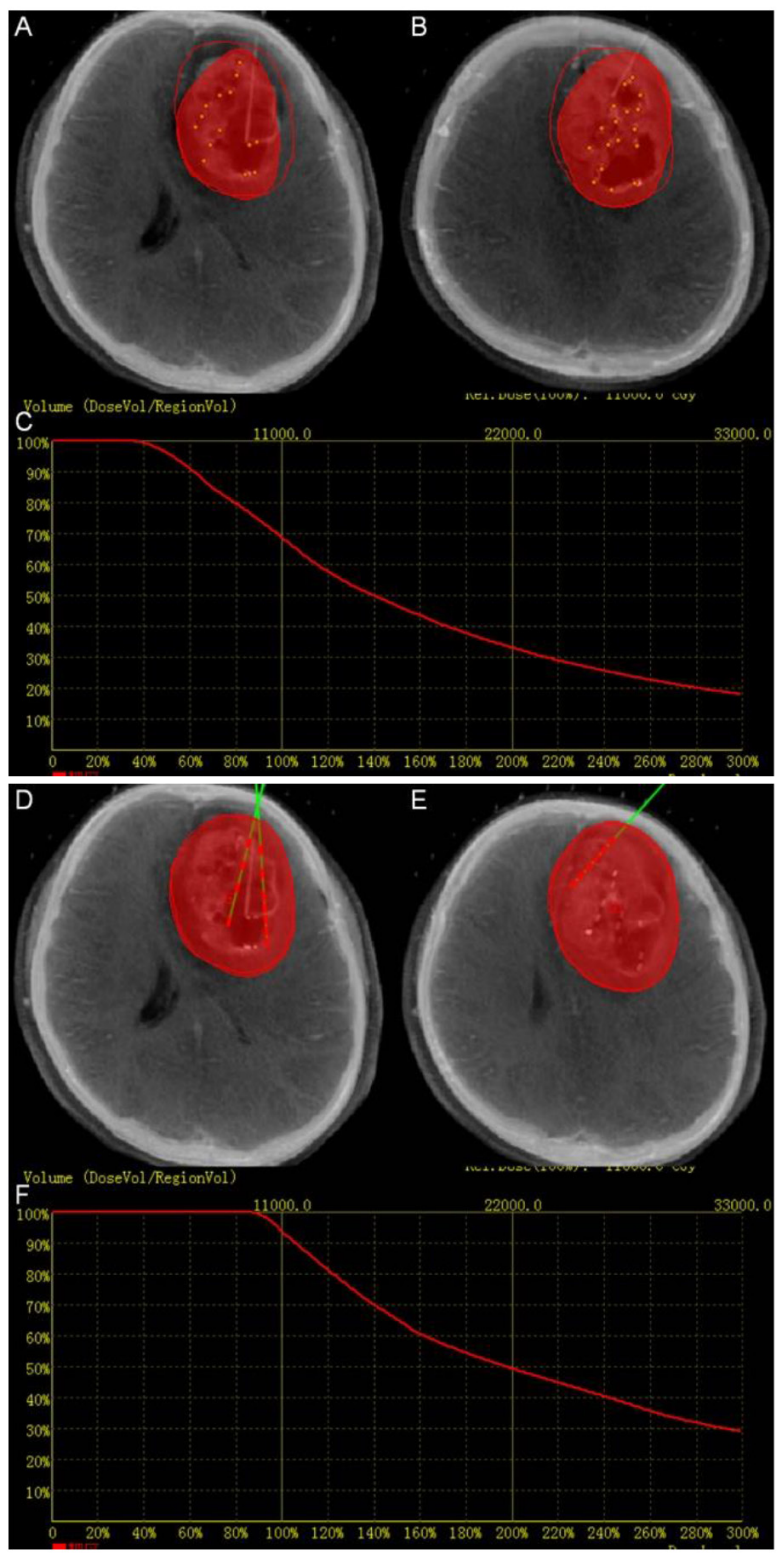
Intra-operation **(A), (B), (D)** and **(E)** Fused image can aid to adjust the position of the catheters and seeds in real time. The red perimeter shows the planned treatment area. The red volume shows the treatment achieved at the stage of the implantation. **(C)** Dose volume histogram of intra-operation. The red curve shows the dose volume histogram of the target. The dose volume showed that the dose did not totally covered the target volume. **(F)** The final dose volume histogram.

**Figure 4:**
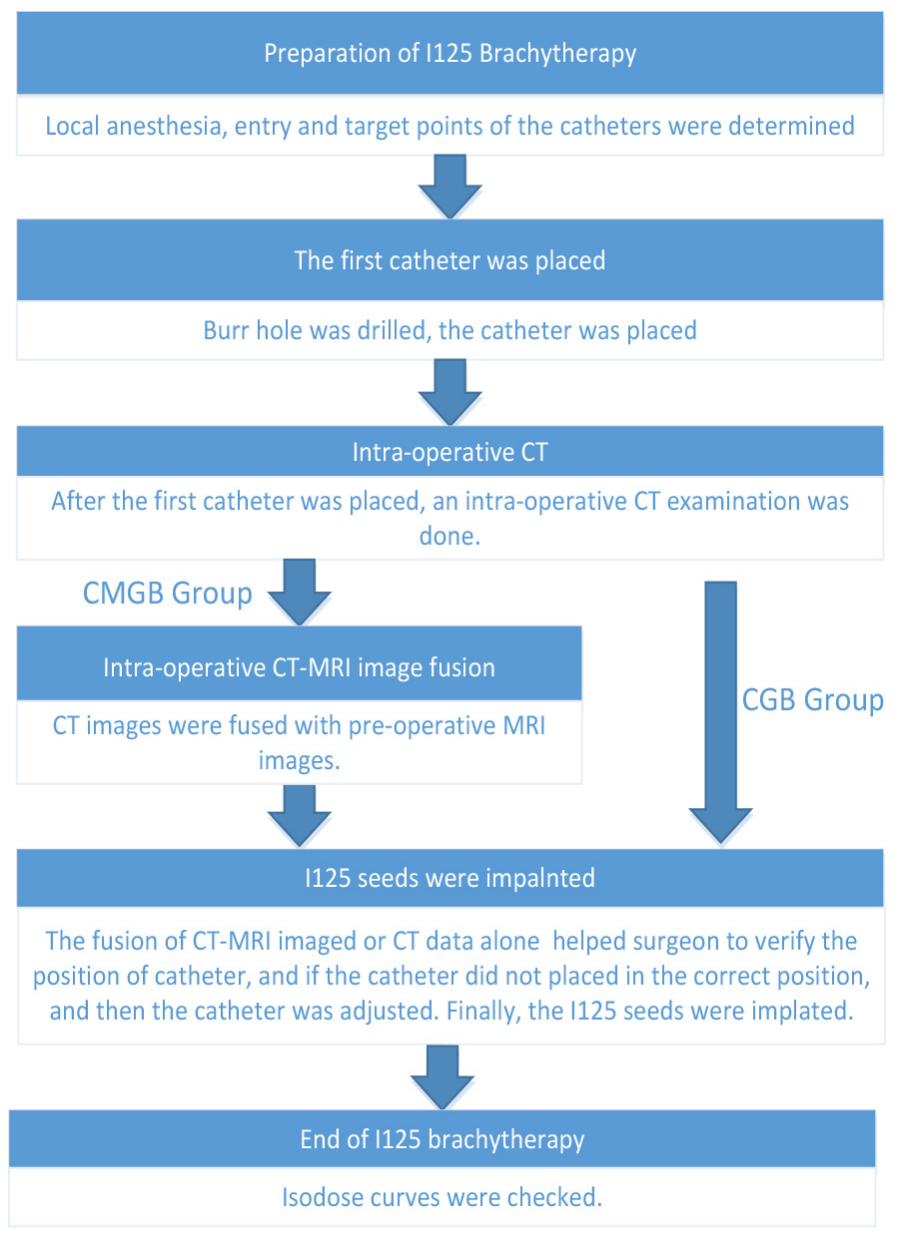
Intra-operation workflow

After the insertion of the first catheter, intra-operative CT images were loaded into fusion platform which we designed and were fused to the pre-operative MRI data. The position of catheter can be seen on the screen. Intra-operative CT-MR images fusion provided the comparison of the planning and the operation. If the catheter did not reach the planned position, it can be corrected until the catheter reached the determined target point, at which time the seeds were implanted (Figure 3). Figure 4 summarizes the intra-operative workflow.

#### Post-operation

After the operation, an evaluation was performed for dosimetry verification and assessment of the position of the catheters and seeds. Intra-operative CT images fused with pre-operative MRI were used to calculate the dose distribution in brain tumors.

### Image fusion

Fusion is defined as an operation where two volumes are registered and resampled into one following a predefined formula. Typical fusions algorithms apply simple weighted image addition, which can be described by the following equation (1):

$$I(x)=C_{1}\cdot I_{1}(x)+C_{2}\cdot I_{2}(x)$$ (1)

Where { x⊂Ω }

where *I(x)* is the value at voxel *x* of fused image inside domain *Ω. I_1_(x)* and *I_2_(x)*represents voxels of two original images. *C_1_* and *C_2_* are weighted constant summing to 1.

In addition to weighted factors, the fusion algorithm we adopt also enables manual adjustment of the window and level of the fused images, to visually emphasis of lesions. The algorithm, separated into two steps, follows the equations (2) (3) below:

$$I_{k}^{'}(x)=\left[I_{k}(x)-\left(l_{k}-\frac{w_{k}}{2}\right)\right]\cdot \frac{I_{max\;}}{w}$$ (2)

$$\text{I}(\text{x})=p(I_{1}^{'}(\text{x}))+p(I_{2}^{'}(\text{x}))$$ (3)

$$\text{where }p(\text{y})=\{\begin{array}{c} {\text{I}}_{\text{max}}\;\;\;for\;y>{\text{I}}_{\text{max}}\\ 0\;\;\;\;for\;y<0\;\;\\ y\;\;\;\;Other\;wise \end{array}$$

where *l_k_* and *w_k_* are level and window setting of the image, respectively. *I_max_* is the maximum grayscale pixel value of the image.

To facilitate segmentation, the fused image is downsampled into the desired number of slices along either axial, coronal or sagittal direction.

### Validation of the dose distribution

The central aim of our analysis was to evaluate the impact of using the fused data on seed distribution and therefore, final treatment volume. The accuracy of catheter targeting and conformity of dose distribution was compared between the two groups. Conformity index (CI) was a valuable method to measure of how well the prescribed dose covered the tumor volume and normal tissue [40, 41].

The CI can be described as follows:

$$CI=\frac{V_{pd}}{TV}\cdot \frac{V_{pd}}{PIV}$$

Where *V_pd_* is the prescribed dose covering the clinical target volume, *TV* is the target volume, and the *PIV* is the prescribed dose covering the volume. The ideal value of *CI* was 1 (range: 0-1).

### Statistical analysis

Standard summary statistics were used to summarize the demographic and observed outcome measures with *p*-values reported. To enable a comparison with treatment planning, the planning target volume (*PTV*) under CT-MRI image fusion and under CT data only were calculated. The *PTV* was evaluated as paired samples that come from the same population, whereas *V_pd_*, *V150, V200* and *CI* were evaluated as unpaired samples that come from different groups. *PTV*, *V_pd_*, *V150, V200* and *CI* were confirmed to be from a non-normal distribution data via the use of Kolmogorov–Smirnov test (*p*<0.05). Therefore, the Wilcoxon signed rank test was used for *PTV*, and Wilcoxon Rank-Sum (Mann–Whitney U) test was used for *V_pd_*, *V150, V200* and *CI*. All statistical analysis was performed using SPSS Statistics version 22.0 (IBM Corp. Released 2013. IBM SPSS Statistics for Windows. Armonk, NY, USA: IBM Corp).

## CONCLUSIONS

The method presented in this study demonstrated that preoperative CT-MRI fusion for treatment planning and intraoperative CT-MRI fusion for dosimetry evaluation of ^125^I seed implantation is feasible. Real-time dosimetry measurements and catheter position adjustments of brachytherapy based on the intraoperative CT-MRI fusion correlated well with treatment planning. The present results suggest that the real-time intraoperative fusion may be helpful in clinical practice of ^125^I brachytherapy for patients with HGG. This application is proposed to overcome the limitations of CT guided brachytherapy and provide an opportunity for correction of potential flaw of the operation. We believe that this study can provide a valuable method to meet dosimetry goals.
